# Direct evaluation of influence of electron damage on the subcell performance in triple-junction solar cells using photoluminescence decays

**DOI:** 10.1038/s41598-017-02141-0

**Published:** 2017-05-16

**Authors:** David M. Tex, Tetsuya Nakamura, Mitsuru Imaizumi, Takeshi Ohshima, Yoshihiko Kanemitsu

**Affiliations:** 1 Institute for Chemical Research, Kyoto University, Uji, Kyoto 611-0011 Japan; 2 Japan Aerospace Exploration Agency, Tsukuba, Ibaraki 305-8505 Japan; 3 National Institutes for Quantum and Radiological Science and Technology, Takasaki, Gunma 370-1292 Japan

## Abstract

Tandem solar cells are suited for space applications due to their high performance, but also have to be designed in such a way to minimize influence of degradation by the high energy particle flux in space. The analysis of the subcell performance is crucial to understand the device physics and achieve optimized designs of tandem solar cells. Here, the radiation-induced damage of inverted grown InGaP/GaAs/InGaAs triple-junction solar cells for various electron fluences are characterized using conventional current-voltage (I–V) measurements and time-resolved photoluminescence (PL). The conversion efficiencies of the entire device before and after damage are measured with I–V curves and compared with the efficiencies predicted from the time-resolved method. Using the time-resolved data the change in the carrier dynamics in the subcells can be discussed. Our optical method allows to predict the absolute electrical conversion efficiency of the device with an accuracy of better than 5%. While both InGaP and GaAs subcells suffered from significant material degradation, the performance loss of the total device can be completely ascribed to the damage in the GaAs subcell. This points out the importance of high internal electric fields at the operating point.

## Introduction

The tandem solar cells utilize several junctions connected in series to achieve high conversion efficiencies^1–3^. Triple-junction solar cells, such as InGaP/GaAs/Ge solar cells, have been mainly developed for use in space^4^. However, due to the proton and electron particle flux, solar cells suffer damage and their performance degrades over time^5^. The damage through radiation is usually very complex^6^. The detailed mechanism of the performance degradation is still unknown and empirical models are used to predict the lifetime of solar cells on specific space missions^5, 7, 8^.

Due to the series constraint in tandem devices, the current is reduced but the voltage of the device is improved. Each subcell has its own operating point, and its change due to radiation damage has to be understood for further improvement^9^. To understand the electrical behavior of the subcells, our newly introduced all-optical technique would be suited^10^. The commonly used empirical methods rely on device characterization with current-voltage (I–V) measurements, or light-biasing for subcell analysis with external quantum efficiency (EQE)^11^. Electroluminescence (EL) measurements^12^ provided important information on the subcell degradation^13^. However, due to the series constraint, the EL signal includes transport and material properties at the same time, with possible non-negligibe influence from neighboring subcells. In contrary, our optical method allows to analyze electrical properties of the subcells directly without need of current injection, contacts, or light-biasing^14^.

Steady-state luminescence signals can be used to analyze the solar cells material quality^15–17^, and time-resolved luminescence signals provide information on the current generation dynamics^10, 14^. Using a detailed bias-voltage dependence, we verified that the time-resolved photoluminescence (PL) decays can be used to evaluate the subcell conversion efficiencies by observing physical parameters such as the drift time constant^18^. By extending this technique to more devices with different device structures, further understanding of the influence of the radiation damage on the current generation dynamics can be gained.

In this work, we measure the I–V curves and PL decays of InGaP and GaAs subcells in ten triple-junction solar cells before and after irradiation with electrons. Inverted grown (growth from top to bottom) triple-junction solar cells with conversion efficiencies of about 30% have been used. From the power dependence of the PL decays, we obtain the optical time constants for recombination in flat-band condition and charge separation in short-circuit and also the point of maximum power. By comparing the optically obtained time constants and electrical parameters, it is shown that our optical method allows to predict the absolute electrical conversion efficiency of the device with an accuracy of better than 5%. Since the carrier dynamics have been measured directly in each subcell, we can discuss the influence of radiation on the current generation process. We find that the recombination losses increased in both InGaP and GaAs subcells due to the electron irradiation. However, due to the high internal electric fields in the InGaP subcell, the material degradation of the InGaP subcell has only a weak influence on the entire device performance at the point of maximum power.

## Results

Ten inverted grown InGaP/GaAs/InGaAs triple-junction solar cells were characterized using I–V measurements and time-resolved PL measurements before and after electron irradiation. The details of the samples and the measurement setup are given in the Methods. The electron fluences are provided in Table 1.

**Table 1:** Overview of samples.

Sample #	1	2	3	4	5	6	7	8	9	10
Fluence (10^13^/cm^2^)	3	3	10	10	30	30	100	100	300	300
* I*_*sc*_ (mA)	67.49	67.30	67.57	67.22	65.59	66.81	65.86	65.48	63.14	63.74
* V*_*oc*_ (V)	2.75	2.74	2.70	2.70	2.66	2.66	2.58	2.58	2.47	2.45
* P*_*m*_ (mW)	151.2	150.3	147.9	144.0	142.3	144.8	136.9	134.6	120.9	116.9
InGaP										
* τ*_1_	0.526	0.444	0.504	0.554	0.493	0.464	0.475	0.458	0.488	0.473
* τ*_2_	11.84	12.13	12.59	16.61	9.92	9.69	7.54	8.17	4.46	4.38
* τ*_3_	3.11	3.50	3.42	3.87	3.00	2.85	2.26	1.36	1.21	2.06
GaAs										
* τ*_1_	0.342	0.352	0.261	0.299	0.259	0.285	0.285	0.301	0.257	0.297
* τ*_2_	6.94	7.38	2.54	2.63	0.985	1.04	0.400	0.381	0.260	0.273
* τ*_3_	1.00	1.03	0.692	0.736	0.546	0.538	0.400	0.381	0.260	0.273

The table shows the irradiation fluence for each sample, and the final I–V parameters and PL decay time constants for the InGaP and GaAs subcells after irradiation.

### I–V measurements

**Figure 1:**
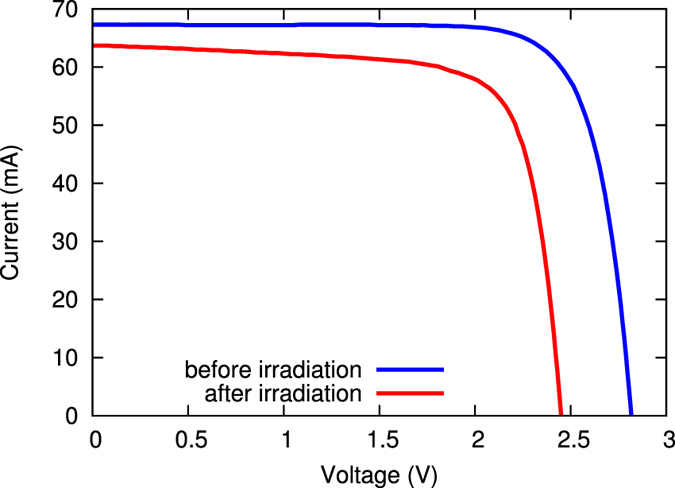
I–V curves of sample #10 before (blue curve) and after (red curve) electron irradiation. Due to strong electron irradiation, both *I*_*sc*_ and *V*_*oc*_ dropped. Also the fill factor changed significantly.

**Figure 2:**
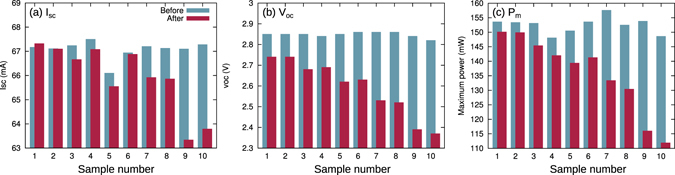
Summary of I–V measurements. The data shown are for (**a**) short circuit current, (**b**) open circuit voltage, and (**c**) maximum power before (blue bars) and after (red bars) electron irradiation.

First the devices were characterized with I–V measurements. One example of I–V curves before and after electron irradiation is shown for sample #10 in Fig. 1. Due to the damage induced by the electron fluence, both short circuit current (*I*_*sc*_) and open circuit voltage (*V*_*oc*_) dropped. The values for *I*_*sc*_, *V*_*oc*_, and the maximum power (*P*_*m*_) after irradiation are shown in Table 1. The initial values (before electron irradiation) are similar to those for samples without or small fluence. The degradation of these values due to electron damage are visualized in Fig. 2(a–c).

As can be confirmed from Fig. 2(a), the initial *I*_*sc*_ values (blue bars) were almost constant 67 mA. Only sample #5 had a 1 mA smaller *I*_*sc*_. The red bars indicate the measured values after electron irradiation. Almost all values decreased as a result of the degradation of the sample by electron induced damage. The *I*_*sc*_ for samples #1 lies slightly above the initial value, which is considered to be within the measurement error.

The trend of *V*_*oc*_ is shown in Fig. 2(b). Compared to *I*_*sc*_, a significant influence of electron damage on *V*_*oc*_ is observed already at weak fluences. The initial maximum power (conversion efficiency) is shown with the blue bars in Fig. 2(c). The red bars show a small degradation for low fluences and a large degradation for high fluences. This trend is quite different than that observed for *I*_*sc*_ and *V*_*oc*_. Another physical factor is required to explain the degradation of the output power.

The degradation of the output power is a result of the change in the subcell operating points. In order to understand which subcell is responsible for the degradation and how each subcell contribute to the new operating point, we characterize the top and middle subcell individually with an optical method.

### PL measurements

**Figure 3:**
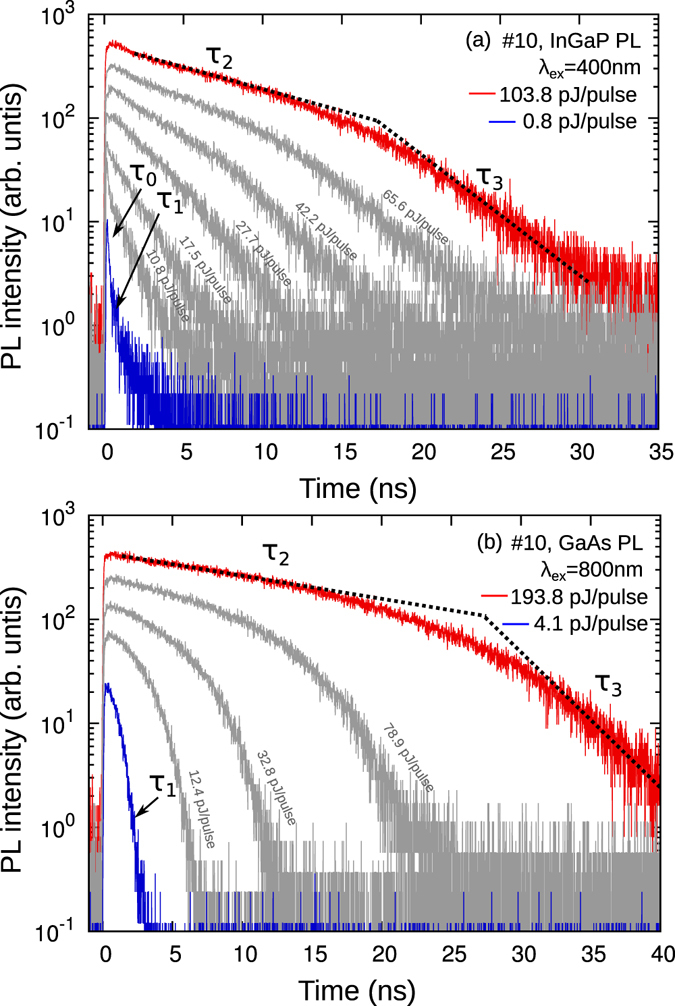
Power dependence of PL decays from (**a**) InGaP and (**b**) GaAs subcell of sample #10, before irradiation.

All PL measurements were performed under open-circuit condition. The power dependence of the PL decays from the InGaP subcell in sample #10 after weak electron damage is shown in Fig. 3(a). The excitation wavelength was 400 nm, and we recorded PL decay curves for excitation powers between 0.8 and 103.8 pJ/pulse. At low excitation powers (blue data) a fast decay curve is observed, which slows down for higher excitation powers (red data). This general trend is similar with those reported previously in experiment and theory^10, 14, 18, 19^.

First we discuss the details of the decay for low excitation power (Fig. 3(a), blue data). Short after excitation an ultrafast initial decay (*τ*_0_) is observed. The decay time constant is faster than 30 ps, which was confirmed with streak camera measurements and assigned to fast carrier trapping, which may be from surface traps. After this decay a fast single exponential decay (*τ*_1_ ≈ 500 ps) is observed. The decay component *τ*_1_ is interpreted as the fast charge separation due to the strong electric field under low carrier densities^10, 14^. We note that in contrary to earlier reports on upward grown structures^14^, the final slow decay at low powers is more pronounced, which indicates a higher luminescence efficiency of the inverted grown InGaP layer.

By increasing the excitation power a slow shoulder emerges, which becomes the dominant decay component at high excitation powers (Fig. 3(a), red data: *τ*_2_ ≈ 10 ns). The saturated value of the slow decay at high powers serves for a clear definition of *τ*_2_^10, 18^. The time constant *τ*_2_ is assigned to the mainly non-radiative carrier recombination time constant under high carrier densities, where the pn-junction is in flat-band condition. The slow decay *τ*_2_ is followed by a faster decay (*τ*_3_ ≈ 3 ns). We consider that this time constant strongly reflects the charge separation due to the internal electric field. The value of *τ*_3_ is defined at that point where the second derivative is zero for the first time after the *τ*_2_ decay. *τ*_3_ is significantly slower than *τ*_1_, which is interpreted as a result of slower charge separation under high carrier densities (reduced electric fields). Since the visibilities of *τ*_0_, the final slow decay, and the initial decay at high powers are strongly sample dependent, only *τ*_1_, *τ*_2_, and *τ*_3_ are considered to be intrinsic to the pn-junction decay behavior.

The power dependence of the PL decays from the GaAs subcell for sample #10 is shown in Fig. 3(b). The excitation wavelength was 800 nm, and we recorded PL decay curves for excitation powers between 4.1 and 193.8 pJ/pulse. The power dependence is very similar to that observed for the InGaP subcell. The three important time constants *τ*_1_ ≈ 300 ps, *τ*_2_ ≈ 7 ns, and *τ*_3_ ≈ 1 ns can be clearly identified.

**Figure 4:**
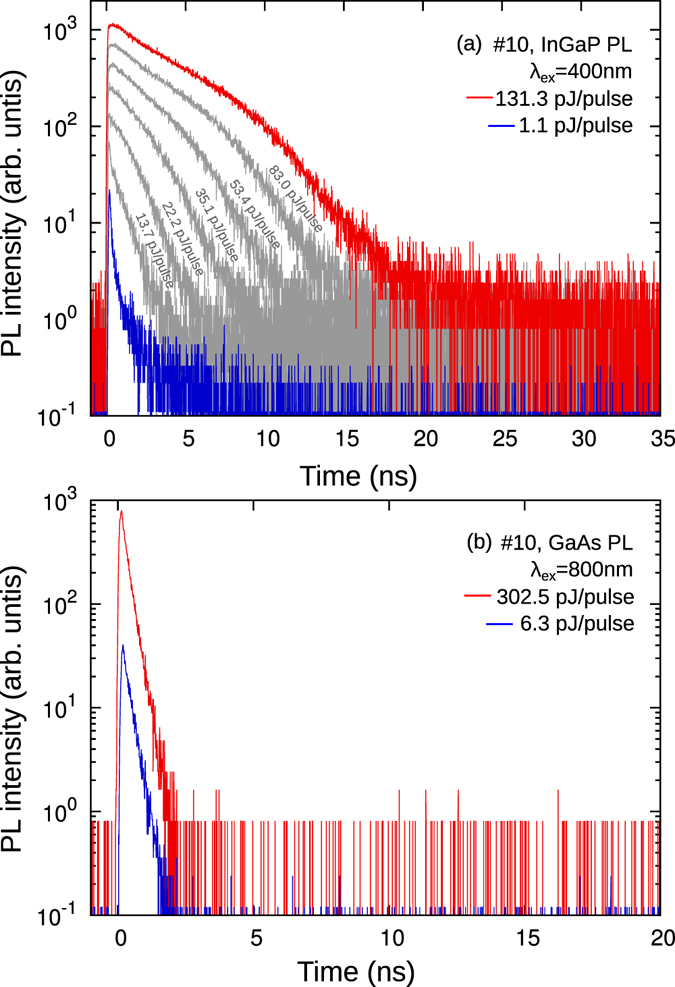
Power dependence of PL decays from (**a**) InGaP and (**b**) GaAs subcell of sample #10, after irradiation.

The above PL decays were for sample #10 with small electron damage. An example of the PL decay curves for sample #10 with high electron damage is shown in Fig. 4(a,b). The time constants *τ*_2_ and *τ*_3_ of the InGaP subcell shown in Fig. 4(a) became smaller, while *τ*_1_ did not change. Meanwhile, the GaAs subcell received much higher damage, since GaAs is weaker against radiation damage^20^. The initial PL intensity signals are comparable to those without damage, although the time constant at high powers (*τ*_2_) became almost a hundred times smaller, now resembling the time constant at low powers (*τ*_1_). This means that the electron damage resulted only in enhancement of the non-radiative recombination path (*τ*_2_) rather than a change in the electric field (*τ*_1_), consistent with results from I–V measurements^6^. For such a strong damage, the assignment of *τ*_3_ becomes difficult, because the physics of the *τ*_3_ decay change. To proceed in such a case we set *τ*_3_ = *τ*_2_. The implications will be discussed later.

**Figure 5:**
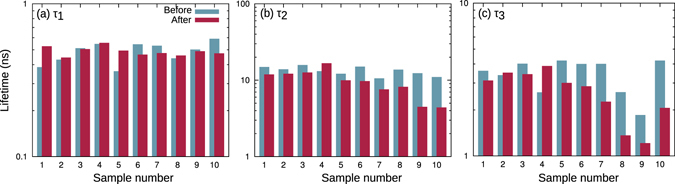
InGaP subcell PL decay time constants: (**a**) *τ*_1_, (**b**) *τ*_2_, and (**c**) *τ*_3_. Blue and red bars represent values before and after electron irradiation, respectively.

All samples showed the power dependence of PL decay curves which was expected from a pn-junction^19^. We measured the time constants of all samples before and after the electron irradiation and the values of the three important time constants *τ*_1_, *τ*_2_, and *τ*_3_ for all samples after irradiation are listed in Table 1. The values before irradiation are very similar to those samples with weak fluence (sample #1 and #2). The graphical comparison of all PL decay time constants before and after irradiation for the InGaP subcell are given in Fig. 5(a–c).

The initial values for the charge separation time constant *τ*_1_ in Fig. 5(a) are shown with the blue bars. After irradiation (red bars) the time constant seems to scatter slightly above and below their initial values, which is considered to be the measurement error. We conclude that *τ*_1_ was almost unaffected by electron irradiation.

The recombination time constant *τ*_2_ of the InGaP subcell is summarized in Fig. 5(b). Overall, the electron irradiation (red bars) lead to a smaller *τ*_2_, meaning a faster nonradiative recombination rate. Significant deviation from the initial value is observed for samples #6–10.

Figure 5(c) shows *τ*_3_ before and after electron irradiation. Especially samples #8 and #9 deviate strongly from the average value. The reason for this is still under investigation, but may have a physical reason rather than just being a pure measurement error. The red bars show that *τ*_3_ is almost unaffected by electron irradiation up to sample #6, and decreases to about half of its value for higher fluences. This correlates well with *τ*_2_, meaning that *τ*_3_ is not only determined by the electric field, but may be also influenced by the recombination rate. To obtain the relative contribution of the electric field, *τ*_3_ has to be related with *τ*_2_^21^.

**Figure 6:**
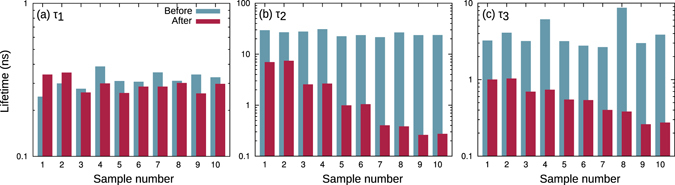
GaAs subcell PL decay time constants: (**a**) *τ*_1_, (**b**) *τ*_2_, and (**c**) *τ*_3_. Blue and red bars represent values before and after electron irradiation, respectively.

The summary of the GaAs time constants is given in Fig. 6(a–c). Figure 6(a) shows that *τ*_1_ stays almost constant, regardless of the damage by the electron fluence. This means that the assignment of *τ*_1_ to the charge separation time constant is valid even for the radiation-weak GaAs device.

The recombination time constant *τ*_2_ was initially almost the same for all samples, as shown with the blue bars in Fig. 6(b). On the other hand, the change due to the electron irradiation (red bars) is remarkable, extending up to two orders of magnitude for the present fluences. This is considered to be a consequence of the weak radiation stability of GaAs^20^.

The initial values of *τ*_3_ are constant for almost all samples, except for samples #4 and 8, as shown in Fig. 6(c) with the blue bars. Unexpected high residual efficiencies have been also confirmed with I–V curves for samples #4 and 8, indicating that these deviations may have real physical meaning and supporting our interpretation that a smaller *τ*_3_ is beneficial for the device. With respect to the data after irradiation (red bars), the degradation trend is similar to that of *τ*_2_ explained above, but a significant deviation from linearity exists, which is important to explain the trend in the maximum power, as shown in the next Section.

### Subcell performance

The carrier collection efficiency at the operating point can be estimated with^14, 18^

1 $${\eta }_{cc}=\frac{{\tau }_{3}^{-1}}{{\tau }_{3}^{-1}+{\tau }_{2}^{-1}}=\frac{1}{1+{\tau }_{3}/{\tau }_{2}},$$

which is a simple relation of *τ*_2_ and *τ*_3_, *i.e*., the competition between extraction as current by the electric field and the loss due to nonradiative recombination. The physical interpretation of *η*_*cc*_ in terms of FF has been given in R﻿ef. 13. Equation 1 assumes that the extraction mechanism is dominant during the *τ*_3_ decay. We note that for heavily damaged samples this may not be the case any more (Fig. 4(b)). In these cases, Eq. 1 serves only as the upper limit. By inserting the time constants obtained in the time-resolved PL measurements, we are able to predict the device performance and compare them with the I–V results. The device conversion efficiency is written as the sum of the subcell conversion efficiencies,

2 $${\eta }_{opt}=\sum {\eta }_{i}{\eta }_{cc,i}.$$

Here, *η*_*i*_ is the theoretically predicted ideal conversion efficiency of the subcells, with contributions of about 19% coming from InGaP, 14% from GaAs and a few percent from InGaAs. Based on the comparison of the I-V curves from triple-junction solar cells with InGaAs and Ge as bottom subcells^13^, we consider that the bottom InGaAs subcell can be neglected in the present analysis. *η*_*cc*,*i*_ is the carrier collection efficiency of each subcell, as defined in Eq. 1. To compare the results with the I–V measurements, we used 136 mW/cm^2^ for the total AM0, 1 sun input power and a 4 cm^2^ large device.

**Figure 7:**
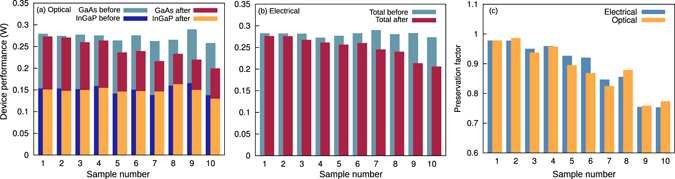
Comparison of optically and electrically obtained solar cell performance. (**a**) Subcell and total device performance predicted using optical measurements. (**b**) Electrically measured output power of the total device. (**c**) Optically and electrically measured preservation factors relating output power of the device before and after irradiation.

The maximum subcell conversion powers predicted by Eq. (2) using the PL decay time constants explained in the previous Section are shown in Fig. 7(a). The InGaP and GaAs subcell performances before irradiation are shown with the light and dark blue bars, respectively. Their intial values are almost constant for all samples. The InGaP and GaAs subcell performances after irradiation are shown with the orange and red bars, respectively. Interestingly, the InGaP subcell performance showed no significant drop for any sample, even though higher nonradiative rates have been confirmed. This means that the electron damage can be compensated by the high electric field existing in the thin InGaP top subcell. The radiation hardness of the InGaP subcell is not only due to the material itself ^20^, but also due to its junction design.

The GaAs subcell performance (Fig. 7(a), red bars) drops significantly for higher electron fluences. The total maximum conversion power predicted by the sum of the optically determined subcell performances can be directly compared with the electrically measured maximum power.

The maximum power of the devices measured by I–V curves before and after irradiation is shown in Fig. 7(b). The blue bars indicate the maximum power before irradiation, and the absolute values are the same as those predicted optically. Also the degradation of the maximum power for higher fluences (Fig. 7(b), red ba﻿rs) correlates well with the values predicted in Fig. 7(a).

A direct comparison of both techniques is given in Fig. 7(c). Here, we obtained the preservation factor of each sample by dividing the maximum output power after irradiation by that before irradiation. Values close to unity mean that the sample suffered almost no damage. The preservation factors predicted using the time-resolved method are very close to the electrically measured factors, within 5%. This confirms that the optical technique presented in this work is an excellent tool to analyze the electrical conversion efficiencies of the subcells.

## Conclusions

We measured the radiation-induced damage of inverted grown InGaP/GaAs/InGaAs solar cells for various electron fluences using I–V measurements and time-resolved PL. All measurements have been performed before and after electron irradiation. The physical meaning of each time constant has been discussed. We find that the conversion efficiency of the entire device measured with I–V curves can be predicted with the time-resolved method with an accuracy of better than 5%. The InGaP and GaAs subcells suffered significant material degradation due to the electron irradiation. However, these increased recombination losses do not influence the performance of the InGaP subcell at the operating point due to its high internal electric fields. Therefore, the performance loss of the entire device can be completely ascribed to the damage in the GaAs subcell. The optical technique is suitable to characterize the electrical performance of the subcells even before electrical contacts have been fabricated.

## Methods

### Samples

Ten inverted grown InGaP/GaAs/InGaAs triple-junction solar cells (Table 1, #1–#10) were characterized using I–V measurements and time-resolved PL measurements. Such tandem cells allow high conversion efficiencies due to inverted metamorphic growth, which allows improved band gap alignment^22^. Our samples were all from the same wafer and had comparable performance. The top InGaP subcell was covered with a contact grid and an anti-reflection coating. Electron irradiation was performed using a Cockcroft-Walton Accelerator from the Japan Atomic Energy Agency. The solar cell was actively water cooled during the irradiation. The electrons were irradiated homogeneously onto the front face with a rate of 1 × 10^12^ cm^−2^s^−1^ and energy of 1 MeV. Since the electrons have high energies, all layers should have received same amount of particle fluence^6, 23^. The electron fluences are provided in Table 1.

### Experimental setup

I–V curves were measured under AM0, 1 sun condition (136.7 mW/cm^2^) at 25 °C. The AM0 spectrum was simulated with a dual source solar simulator (WACOM WXS-130S-L2HV). The time-resolved PL measurements were performed using a mode-locked Ti:sapphire laser for excitation (*λ*_*ex*_ = 800 nm; repetition rate: 8 MHz; pulse duration: ≈200 fs) and a Silicon avalanche photo-diode (APD) for detection. The response function of the APD was double-exponential, beginning with a 60 ps decay for one order of magnitude, and then 230 ps. The important time constants are well above this limit, and therefore they were extracted from the data without deconvolution. The fast decays were confirmed with streak camera measurements.

The PL peak wavelength of the GaAs subcell was about 890 nm, whereas that of InGaP was about 660 nm. For separate excitation of the subcells, we used *λ*_*ex*_ = 400 and 800 nm and the detection wavelength was chosen with an appropriate long- and short-pass filter set. The *λ*_*ex*_ = 400 nm light was obtained via second-harmonic generation in a beta-barium borate crystal. The excitation spot size was ≈150 *μ*m. All PL measurements were performed at room temperature and under open-circuit conditions.
